# Are Sex Drive and Hypersexuality Associated with Pedophilic Interest and Child Sexual Abuse in a Male Community Sample?

**DOI:** 10.1371/journal.pone.0129730

**Published:** 2015-07-06

**Authors:** Verena Klein, Alexander F. Schmidt, Daniel Turner, Peer Briken

**Affiliations:** 1 Institute for Sex Research and Forensic Psychiatry, University Medical Center Hamburg-Eppendorf, Hamburg, Germany; 2 University of Luxembourg, Institute for Health and Behaviour, Integrative Research Unit: Social and Individual Development (INSIDE), Health Promotion and Aggression Prevention, Walferdange, Luxembourg; University of Vienna, School of Psychology, AUSTRIA

## Abstract

Although much is currently known about hypersexuality (in the form of excessive sexual behavior) among sexual offenders, the degree to which hypersexual behavior is linked to paraphilic and especially pedophilic interests in non-forensic populations has not been established. The purpose of the present study was to elucidate the associations between total sexual outlets (TSO) and other sex drive indicators, antisocial behavior, pedophilic interests, and sexual offending behavior in a large population-based community sample of males. The sample included 8,718 German men who participated in an online study. Hypersexual behavior as measured by self-reported TSO, self-reported sex drive, criminal history, and pedophilic interests were assessed. In moderated hierarchical logistic regression analyses self-reported contact sexual offending against children was linked to sexual fantasizing about children and antisociality. There was no association between aggregated sex drive, and sexual abusive behaviour in the multivariate analyses. In contrast, self-reported child pornography consumption was associated with sex drive, sexual fantasies involving children, and antisociality. Nevertheless, in clinical practice an assessment of criminal history and pedophilic interests in hypersexual individuals and vice versa hypersexuality in antisocial or pedophilic men should be considered as particularly antisociality and pedophilic interest are important predictors of sexual offending against prepubescent children.

## Introduction

In recent years, there has been an increasing amount of literature providing empirical evidence for a link between hypersexuality and paraphilic interests in sexual offenders [1, 2]. Hypersexuality is used as an umbrella term to describe excessive sexual behavioral pattern in research and clinical practice. Kinsey et al. [3] coined the term “total sexual outlets/week” (TSO) in order to assess the frequency of sexual behavior. TSO was defined as “the sum of the orgasms derived from the various types of sexual activity in which that individual had engaged” ([3], pp. 510–511) during a week including sexual behaviors such as sexual intercourse and masturbation. Typically, TSO is characterized by a left skewed distribution and shows a peak for the age group between 15 and 25 years. Furthermore, TSO seems to be testosterone dependent and relatively independent from the individual relationship status [4]. In earlier research, Kafka [5] proposed seven orgasms per week over a period of six months as a criterion for hypersexual behavior. Further part of the definition was spending at least 1–2 hours per day with sexual behavior.

Possible prevalence estimations used the TSO/week ≥ 7 cut-off as a behavioral indicator for hypersexual behavior in non-clinical community samples of males. In the Kinsey et al. [3] study 7.6% out of 5,300 men reported a TSO/week ≥7 over the last five years. Masturbation was the most frequently reported sexual practice in the sample. Atwood and Gagnon [6] found that 5% of male high-school students and 3% of male college students masturbated once a day (*N* = 1,077). A large survey on sexual behavior in the United States identified daily masturbation in 3.1% of the male sample (*N* = 3,159). Furthermore, 7.6% of the men reported sexual intercourse at least four times a week [7]. In a population-based Swedish community sample (*N* = 2,450), 12.1% of male participants were identified as being hypersexual [8]. In the latter study high rates of impersonal sexual activity were associated with health problems such as substance abuse and gambling as well as with paraphilic interests in terms of voyeurism, exhibtionism, sadism, and masochism.

In sexual offender populations paraphilic interest has been meta-analytically established as the most important risk factor [9, 10]. Hypersexuality (or sexual preoccupation, high sex drive) is also found among the most important risk factors for sexual offending [11] and was identified as a possible contributing risk factor for sexual and violent reoffending in sexual offenders [12]. Furthermore, hypersexual behavior patterns seem to be more likely in sexual offenders than in community controls [13, 14]. In addition, high sex drive has been reported to be associated with sexual coercive behavior against women [15]. Pornography consumption understood as a behavioral pattern possibly related to hypersexual behavior was associated with recidivism in a sample of 341 high-risk sexual offenders against children. Additionally, sexual deviant content of pornography was a risk factor for reoffending in this sample [16]. One obvious limitation to most of these studies is the fact that they were based on exclusive samples of sexual offenders. However, in a young Swedish community-sample sexual preoccupation (defined as sexual lust almost all time) was identified as risk factor for self-reported sexual coercive behavior [17]. Notably, research on sexual aggression and hypersexual behavior is not altogether consistent. In a study by Malamuth et al [18] sexually aggressive men against females reported a higher preference for impersonal sexual activity (e.g. masturbation frequency, attitudes toward causal sex) but did not indicate higher frequency of orgasms per week and sexual intercourse. Hence, high sex drive did not contribute to sexual aggression in their sample. To the best of our knowledge, the study by Långström and Hanson [9] is the only study demonstrating an association between hypersexuality and paraphilic interests in a community sample.

### Present Research

Our research question connects the findings about hypersexuality among sexual offenders to the link between hypersexual behavior and paraphilic, especially pedophilic interests, in non-forensic populations. Therefore, the first aim is to explore the association between pedophilic sexual interests/ sexual offending behavior and TSO/sex drive indicators in a large population-based community sample of males. Furthermore, due to a lack of research in community samples, only little is known about possible criminological factors and their putative associations with hypersexual behavior in males. Therefore, the second purpose of the present study was to elucidate the association between TSO, other sex drive indicators and antisocial behaviour including sexual offending against children. Moreover, most studies on TSO in community samples have neglected the amount of time spent with sexual fantasies and urges [1]. Thus, the present study also aimed to examine the relationship between TSO and the amount of time spent with sexual fantasizing and pornography consumption.

## Materials and Methods

The reported data are part of a large population-based online study on German males’ sexual interest in prepubescent children [19]. The study was part of a research project funded by the German Federal Ministry of Family Affairs, Senior Citizens, Women, and Youth. A German market research institution was authorised to collect data via an online panel. Participants were informed beforehand in an email about the topic of the study. They provided an online consent form at the beginning of the survey by clicking the online consent form “accept” button. In addition, it was possible to withdraw from the study at any point simply by leaving the survey´s web page. At the end of the survey an option was offered that prevented individual data from being included into the analyses. Complete anonymity and confidentiality was assured to potential participants. Therefore, a university server was used to store the collected data whereas a separate server coded participation status in order to ensure participant compensation via the market research institution. Furthermore, due to this procedure, it was impossible to identify individuals in case legal authorities intended to prosecute men who admitted criminal behavior. The participants were informed about this procedure so that they might answer honestly and received a monetary reward of 20 €. The ethics committee of the German Psychological Society approved the study protocol and the consent procedure.

In total, 17,917 men (≥ 18 years of age) were contacted by the market research institution in order to be representative of the German male population in terms of age and education levels. In consequence, the link has been accessed 10,538 times and data were collected for 10,045 participants. Because of missing data in the individual surveys the effective sample was reduced to 8,718 participants (48.7% of the initially contacted men; 82.7% of participants actually accessing the link). Participants’ mean age was 43.5 years (*SD* = 13.7, range 18–89). Concerning their professional status most of the participants were employed (71.5%, *n* = 6,179) or retired (13.1%, *n* = 1,143), 5.6% (*n* = 488) of the participants were unemployed and 9.7% (*n* = 836) were in professional training at the time of data collection. The majority of participants (56.4%, *n* = 4,874) had a school leaving examination taken at the end of the 13^th^ year, 30.3% (*n* = 2,618) finished school with a high-school diploma, 12.7% (*n* = 1,104) with a secondary modern school qualification, 0.3% (*n* = 24) had no graduation, and 0.3% (*n* = 28) were still in school. Participants differed from the German male population in terms of age and education levels as there was an overrepresentation of higher education and the age range of 30–49 whereas lower education and men over 65 were underrepresented [19]. For detailed results concerning self-reported prevalences of sexual interest in prepubescent children refer to [19].

### Measures

TSO was measured with the following question: “*Please think of a typical week in the last year*: *How many orgasms did you have on average no matter how the orgasm was achieved (e*.*g*., *masturbation*, *sexual encounters*, *wet dreams*)?”. In addition, sex drive (“*Please think of a typical week in the last year*: *How strong was your desire for sexual activity?*”) and the amount of time spent with sexual fantasies, sexual urges, and sexual behavior (“*Please think of a typical day in the last year*: *Please estimate the amount of time you spend with sexual fantasies*, *sexual urges*, *and sexual behavior*.”) as well as with pornography consumption (“*Please think of a typical day in the last year: Please estimate the amount of time you spend viewing pornography (e.g., naked genitals) in order to be sexually aroused?*”) were assessed. Sex drive was rated on a 100-point slider scale. The amount of time spent with sexual fantasies, sexual urges, and sexual behavior as well as with pornography was assessed using an open answer format (hours and minutes per day). Sexual fantasies and behaviors directed at prepubescent children were assessed with a shortened 12-item version of the Explicit Sexual Interest Questionnaire (ESIQ) [20]. The ESIQ has been shown to be a reliable and valid measure of adult and pedophilic sexual interests [20–22]. The items of the shortened version referred to four sexual target categories (prepubescent boys or girls ≤ 12 years and women or men) and consisted of each three items describing sexual fantasies (“*I find it erotic to see a …’s body through the clothes*”, “*I get excited when I imagine that a … stimulates me*”, “*I find it erotic to imagine having sex with a …”*) and sexual behaviors (“*I have sexually caressed a…*”, “*I have tongue kissed a …*”, “*I have enjoyed getting my private parts touched by a …*”). Participants had to indicate on a dichotomous scale (true/false) whether they had experienced the corresponding sexual fantasies and behaviors as adults (> 18 years). The reliability (internal consistency) of the aggregated ESIQ subscales was good: sexual fantasies involving girls (α = .81), boys (α = .86), women (α = .90), and men (α = .92). Child sexual fantasy items were used as indicators of pedophilic interest whereas child sexual behavior items were used as indicating sexual offenses against children. Child pornography consumption was assessed with the following item: “Have you ever watched pornographic depictions of children, e.g., the nude genitals of children, to get sexually aroused after you were 18 years of age?” [true/false]). Again, children were anchored to represent prepubescent stages of sexual maturity. In order to examine participants’ antisocial behavior and criminal history they were asked to answer the following three forced-choice questions: 1. *Have you ever been convicted of an offense against property (etc*. *larceny*, *burglary)?*; 2. *Have you ever been convicted of a violent offense (etc*. *bodily injury)?*; 3. *Have you ever been convicted of a sexual offense (etc*. *sexual coercion*, *rape*, *sexual abuse*)?

### Statistical Analyses

In order to robustly identify outliers the *median absolute deviation* (MAD) [23] was calculated for TSO, amount of time spent with sexual fantasies, urges, and behavior as well as amount of time watching pornography. The MAD analyses yielded cut-offs for outliers of TSO ≥ 10, ≥ 165 minutes for daily sexual fantasies, urges, and behaviors, as well as ≥ 95 minutes for daily pornography consumption. Correlational analyses were conducted to verify the association between absolute TSO (i.e., as a dimensional construct), subjective sex drive, amount of time spent with sexual fantasies and viewing pornography. Further correlations were calculated to examine the relationship between TSO or sex drive indicators and pedophilic interests, sexually offending behavior, and criminal history. In order to elucidate the impact of the categorical cut-off for hypersexuality, participants were divided in two groups: low and high self-reported hypersexuality as based on the proposed cut-off value TSO ≥ 7 by Kafka [5]. Because frequency of sexual activity and sex drive on average declines with age in so far as younger individuals report more sexual outlets per week [7] we conducted additional analyses utilizing partial correlations controlled for age. Finally, we conducted moderated hierarchical binary logistic regression analyses [24] to test for possible interaction effects of sex drive indicators, antisociality, and child-related sexual fantasies on contact child sexual abuse and child pornography use.

## Results

Overall, the mean TSO/week was 3.46 (*SD* = 2.29). On average the participants spent 45.2 minutes/day (*SD* = 38.1) with sexual fantasies and urges. The mean score of sex drive was 59.7 (*SD* = 21.4) and the reported daily duration for consuming pornography was 13.1 minutes (*SD* = 19.3). The non-hypersexual group consisted of 7,339 males (87.9%), whereas 1,011 males (12.1%) were classified into the hypersexual group according to the classical cut-off value TSO ≥ 7. Sex drive and TSO positively correlated with time consuming sexual fantasies and urges. Furthermore, a significant positive correlation occurred between TSO and sex drive with the amount of time spent with pornography consumption. Partial correlations corrected for possible age and education effects showed a very similar pattern of results (see Table 1). As all measures indicating sex drive were positively intercorrelated we calculated an aggregated sex drive index consisting of *z*-standardized TSO/week, subjective sex drive ratings, as well as amount of time spent viewing pornography and fantasizing about sexual content (α = .66). In addition, an antisociality index was determined by aggregating self-reported preconvictions (violent, property, sexual). As sexual preconvictions could overlap with self-reported sexual victimization of children we also calculated an aggregated antisociality index leaving out sexual preconvictions.

**Table 1:** Overview of sex drive intercorrelations (above diagonal zero-order correlations, below diagonal partial correlations corrected for age and education).

	1	2	3	4	5	6
Age	**-.28**	**-.14**	**-.15**	**-.12**	**-.15**	**-.24**
Education^a^	**.06**	.02	**.06**	-.02	-.02	.02
1. TSO (absolute value)	-	**.74**	**.42**	**.30**	**.25**	**.72**
2. Hypersexuality Group^b^	**.72**	-	**.21**	**.15**	**.15**	**.47**
3. Sex Drive	**.41**	**.19**	-	**.37**	**.24**	**.74**
4. Time spent with sexual fantasies	**.27**	**.13**	**.36**	-	**.39**	**.74**
5. Time spent with pornography consumption	**.23**	**.15**	**.21**	**.37**	-	**.68**
6. Aggregated Sex Drive Index	**.69**	**.43**	**.72**	**.72**	**.64**	-

Note

^a^ Higher values indicate at least high-school diploma level;

^b^ Higher values indicate TSO ≥ 7 (Hypersexuality). Bold correlation coefficients *p* < .05.

### Zero-order correlations

In order to verify the relation between TSO and pedophilic interests, correlation analyses were conducted. Sex drive, TSO, and TSO ≥ 7 were positively associated with sexual fantasies involving children and child pornography consumption. Additionally, aggregated sex drive positively correlated with self-reported sexual offending behavior in the past. Concerning antisocial behavior, TSO and TSO ≥ 7 were positively related to a history of property and violent offences in the past, whereas no association with sexual offending was found. Aggregated sex drive showed a positive correlation with all offending categories. However, effect sizes were small (Table 2).

**Table 2:** Overview of zero-order child sexual abuse risk factor intercorrelations.

	1	2	3	4	5	6	7	8	9
Age	.01	-.02	-.01	-.01	-.00	**-.02**	**-.05**	**-.04**	**.40**
Education^a^	**-.09**	**-.09**	-.01	**-.11**	**-.11**	**-.02**	-.01	-.01	**-.07**
TSO (absolute value)	**.03**	**.03**	.01	**.04**	**.04**	.00	**.07**	**.08**	**-.10**
Hypersexuality Group^b^	**.03**	**.03**	.01	**.04**	.02	.00	**.07**	**.07**	**-.08**
Sex drive	.02	**.04**	**.02**	**.04**	**.04**	**.04**	**.06**	**.10**	-.01
Time spent with sexual fantasies	**.05**	**.07**	**.04**	**.08**	**.08**	**.07**	**.06**	**.08**	**-.02**
Time spent with pornography consumption	**.06**	.02	**.03**	**.06**	**.05**	**.08**	**.12**	**.14**	**-.12**
Aggregated Sex Drive Index	**.06**	**.06**	**.04**	**.08**	**.07**	**.07**	**.11**	**.15**	**-.09**
1. Prior conviction violent offending (*n* = 304)	-	**.26**	**.21**	**.74**	**.74**	**.14**	**.07**	**.07**	.00
2. Prior conviction property offending (*n* = 193)		-	**.13**	**.82**	**.84**	**.08**	**.07**	**.05**	.01
3. Prior conviction sexual offending (*n* = 36)			-	**.42**	**.21**	**.32**	**.22**	**.23**	.00
4. Aggregated Antisociality Index				-	**.98**	**.20**	**.13**	**.12**	.01
5. Aggregated Antisociality Index (w/o sex. offences)					-	**.14**	**.09**	**.08**	.01
6. Contact sexual offending against children (*n* = 132)						-	**.36**	**.44**	**-.03**
7. Child pornography (n = 209)							-	**.50**	**-.06**
8. Aggregated Pedophilic Fantasies (Maximum)								-	**-.06**
9. Ever lived with a lover for at least two years (*n* = 7115)									-

*Note*. Bold correlation coefficients *p* < .05.

^a^ Higher values indicate at least high-school diploma level;

^b^ Higher values indicate TSO ≥ 7 (Hypersexuality).

### Logistic regression analyses

**Fig 1:**
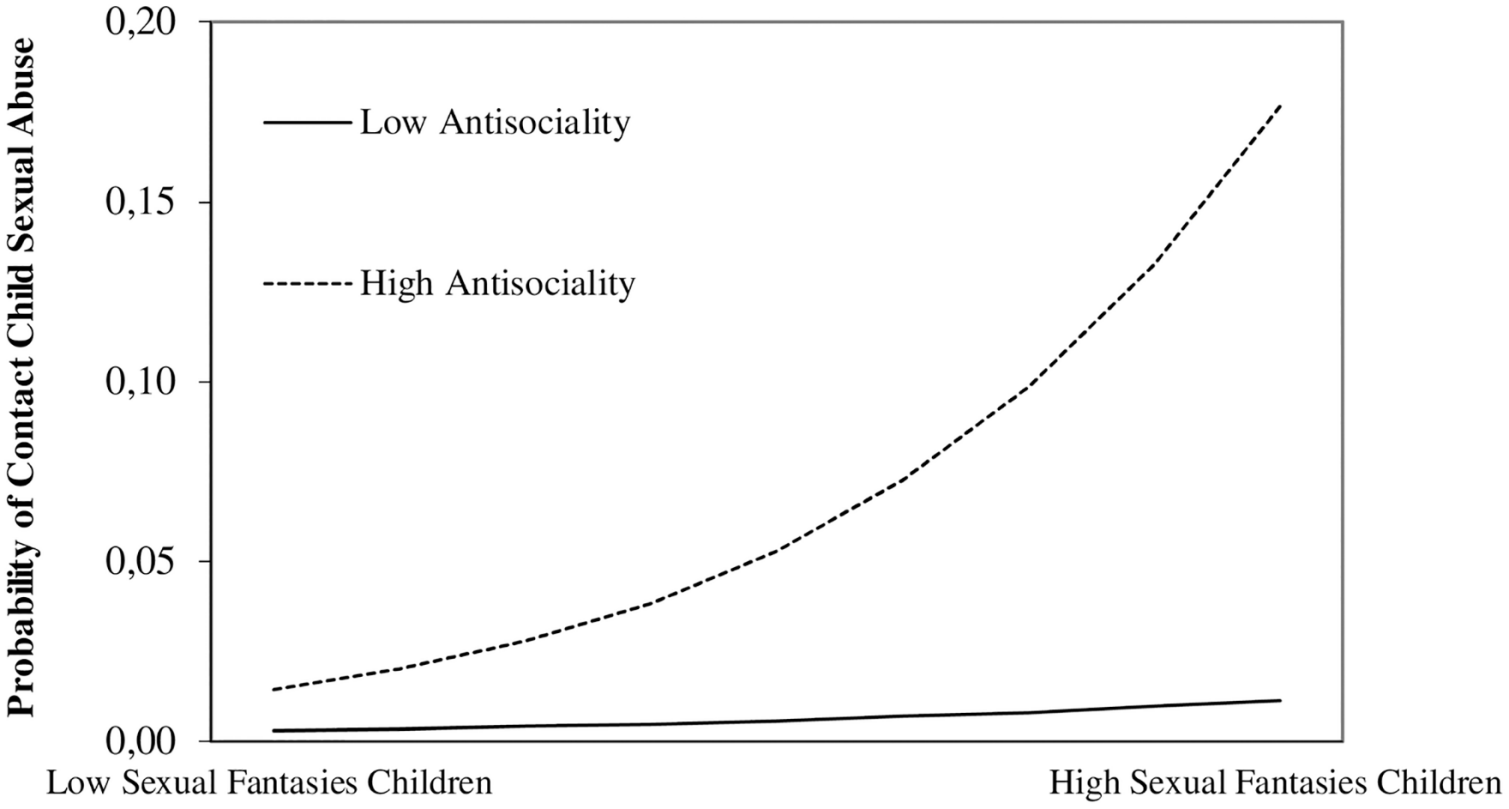
Probability of contact child sexual abuse as a function of self-reported amount of child sexual fantasies (+ 1 *SD vs*. - 1 *SD*) and antisociality (aggregated non-sexual preconvictions; average of the sample [low] vs. two different preconvictions [high]).

Hierarchical logistic regression analyses revealed that self-reported contact sexual offending against prepubescent children was associated with child sexual fantasies and antisociality (without sexual preconvictions). In addition, a significant interaction between antisociality and sexual fantasies involving children emerged corroborating a moderation effect (Fig 1): For men who reported no preconvictions in the sample no link between sexual fantasizing about children and contact sexual offending against children emerged. However, the likelihood to report contact sexual abuse of children significantly increased for men who had reported prior convictions for criminal offences from two different categories (violence, property). Notably, aggregated sex drive showed no association with contact sexual abuse and no further interaction effects emerged (Table 3). Similar logistic regression analyses with self-reported child pornography consumption as criterion identified three independent links to sex drive, sexual fantasies involving children, and antisociality excluding sexual preconvictions. No further interactions were revealed.

**Table 3:** Summary of hierarchical logistic regression analyses for child sexual abuse as a function of sex drive, antisociality, and sexual fantasies involving children.

	Contact Child Sexual Abuse				Child Pornography Use			
Predictor	*R* ^2^	ß	Exp(ß)	*CI*_95%_	*R* ^2^	ß	Exp(ß)	*CI*_95%_
Step 1	.37***				.37***			
Sex Drive (SDR)		.11	1.11	0.91–1.36		.35***	1.41	1.20–1.67
Antisociality (AS)		.39***	1.47	1.33–1.63		.21***	1.24	1.12–1.37
Sexual Fantasies Children (SFC)		.67***	1.96	1.83–2.10		.69***	2.00	1.88–2.13
Step 2	.38***				.38***			
SDR		.24	1.27	0.96–1.67		.43***	1.53	1.25–1.87
AS		.29***	1.34	1.16–1.55		.18*	1.20	1.03–1.39
SFC		.69***	1.99	1.84–2.15		.71***	2.03	1.88–2.18
SDR x AS		-.10	0.90	0.79–1.03		-.11	0.90	0.79–1.02
SDR x SFC		-.04	0.97	0.91–1.03		-.03	0.97	0.92–1.03
SFC x AS		.12**	1.13	1.04–1.22		.06*	1.07	1.00–1.14
Step 3	.39***				.38***			
SDR		.23	1.26	0.96–1.66		.43***	1.53	1.25–1.87
AS		.28***	1.33	1.13–1.56		.18*	1.20	1.04–1.37
SFC		.69***	2.00	1.84–2.16		.71***	2.03	1.88–2.18
SDR x AS		-.04	0.96	0.83–1.11		-.12	0.88	0.77–1.02
SDR x SFC		-.04	0.96	0.90–1.32		-.03	0.97	0.92–1.03
SFC x AS		.17**	1.19	1.07–1.32		.05	1.06	0.98–1.14
SDR x AS x SFC		-.06	0.93	0.88–1.01		.01	1.02	0.95–1.08

*Note*. *N* = 8595;

*** *p* < .001;

** *p* < .01;

* *p* < .05

Although, the moderation explained statistically significant shares of criterion variance the net increments were practically irrelevant as they accounted for only a 1% increase in explained variance. However, the independent multivariate main effects ranged between odds ratios from 1.1 to 2.0 (Table 3) for antisociality, sexual fantasies involving prepubescent children, and sex drive (the latter only in case of self-reported child pornography use).

## Discussion

The present study provides clinically relevant insight into the actual paraphilic and criminological correlates of hypersexual behavior in a large non-clinical male community sample. Etiological models and theories on sexual offending against children consider paraphilic sexual interest and antisociality as import contributing risk factors for sexual abusive behavior [9, 25]. The present results are in line with this notion. In multivariate statistical analyses antisocial behavior and sexual fantasies involving children, an indicator for paraphilic interests, were associated with contact child sexual abuse. Moreover, the significant interaction between antisociality and sexual fantasies involving children in logistic regression analyses may indicate that the probability of contact sexual abuse markedly increases in men with particularly high rates of self-reported amount of sexual fantasies involving children and antisocial behavior in the past. In contrast, neither sex drive as such nor in combination with pedophilic fantasies showed an association with contact sexual abuse. Hence, the present results indicate that the effects of sex drive in general and specifically hypersexual behavior as measured by the TSO on self-reported contact sexual abusive behaviour with children are rather small on the level of zero-order correlations and completely vanish once entered into multivariate analyses.

Current research provides evidence for child pornography consumption as predictor for sexual interest in children [26]. Among pornography users, Ray et al [27] found that child pornography users were also more likely to report interest in sexual contact with children. Furthermore, an association between child pornography consumption and sexual coercive behavior was identified in a sample of young Scandinavian men [28]. Consistent with previous results, in the present sample child pornography consumption was positively related to contact sexual offending against children and sexual fantasies involving children. In addition, aggregated sex drive, antisocial behavior, and sexual fantasies involving children were identified as risk factors for child pornography consumption. Hence, for all three constructs there seem to be substantial independent links to child pornography consumption. In the literature several explanations for child pornography consumption are discussed. The underlying motivational aspects to engage in the consumption of child pornography seem to be sexual interests in children and/or thrill-seeking behavior as a result of habituation to mainstream pornography [27, 28]. Pornography dependence is a common sexual behavioral pattern in hypersexual men [1, 2]. Therefore, the association between sex drive and child pornography consumption may be explained by the fact that frequent pornography consumption and a wide-spread interest in all sorts of (a)typical pornography can be seen as indications of increased sex drive. Accordingly, in a sample of male juveniles, Svedin et al [29] identified a relationship between frequent pornography use and child pornography consumption. Hence, it seems possible that persons who frequently engage in pornography consumption have an increased risk of getting in contact with child pornography [30]. Similarly, in a further study the variable frequent sexual lust predicted child pornography use [28]. Still, it remains unclear whether sex drive leads to pornography use, or vice versa. Also a circular reinforcing process in which the availability of pornography on the internet serves as a strong reinforcer for hypersexual behavior seems possible. Therefore, a non-correlative (i.e., ideally longitudinal) examination of the causal extent to which hypersexual behavior/sex drive impacts the use of atypical pornography use should be aimed for in future research.

The known association between antisocial behavior and testosterone [31] has not been investigated in its relationship with sexuality related aspects. In the present study a relation between high TSO and indicators for antisociality were found. However, these findings are limited by small effect sizes. Future research should therefore examine more closely the interplay between the level of testosterone, TSO, and antisociality.

### Limitations and Outlook

There is a lack of research on the association between the amount of time spent with sexual fantasies and urges with TSO in community samples [1]. In the present study, TSO and sex drive were associated with higher rates of time consuming sexual fantasies and pornography use. This finding was expected and suggests that the amount of time spent with sexual activities may be important for the definition of hypersexual behavior [5]. Nevertheless, for the definition of a clinical disorder not only symptomatic behavior but psychological distress and/or maybe also the criterion of causing harm to non-consenting others should be taken into concern. In the consequence, the present study is limited by the lack of information on clinically relevant characteristics caused by hypersexual behavior or high sex drive, even if the potential criterion causing harm to non-consenting others was considered. Further research on hypersexual behavior should address clinical distress as criterion beside the amount of orgasms and time spending with sexuality-related issues.

Several limitations of this study need to be acknowledged. First of all the data are based on self-report and results are limited to the German population. Moreover, the effect sizes in statistical analyses particularly in the interaction terms were rather small. This study was also limited by its cross-sectional correlational design. In addition, it is important to note that in the present study hypersexual behavior and sex drive were based on self-report and should not be confused neither with constructs which are used in sexual offender risk assessments such as sexual preoccupation in the Stable-2007 [32] nor with the diagnostic criteria of hypersexual disorder [1]. Furthermore, questions about total sexual outlets, sex drive, and sexual fantasies/urges were asked in the form of "a typical week or typical day in the past year". This kind of formulation could be more vulnerable to recall bias than asking about the past week, where the week is "randomly selected" and thus might be construed as more representative of the past year. Also the operationalization of antisocial behavior as preconvictions can be deemed a rather conservative criterion. Further studies could measure antisocial behavior by asking if the person had ever stolen, committed assault, or other antisocial acts. Another aspect to be added to future research is the distinction between intercourse/sexual activity within relationships and impersonal sexual activity. This can be hypothesized to be particularly important, because sexual activity in a stable relationship on average is associated with positive mood whereas high rates of impersonal sexual activity are often related to negative mood states [7, 8]. In order to examine possible intimacy problems that may correlate with hypersexuality, further studies, which take the proposed distinction between sexual outlets into account, will need to be undertaken [2].

The results of the present study suggest that the association between hypersexual behaviour as measured by the TSO, sex drive, and contact sexual abusive behaviour in our community sample of men was lower than expected. In contrast, an association occurred between sex drive indicators and child pornography consumption. An implication of these findings is that in the assessment of hypersexual individuals atypical pornography consumption should be taken into account. Nonetheless, in clinical practice (and particularly in forensic populations) an assessment of criminal history and pedophilic interests in hypersexual individuals and vice versa hypersexuality in antisocial or pedophilic men should still be considered.
