# Correction: Are Sex Drive and Hypersexuality Associated with Pedophilic Interest and Child Sexual Abuse in a Male Community Sample?

**DOI:** 10.1371/journal.pone.0139533

**Published:** 2015-09-28

**Authors:** The PLOS ONE Staff

An error in the last sentence of the Abstract occurred during the production process. The publisher apologizes for the error. Please see the correct Abstract text here:

Although much is currently known about hypersexuality (in the form of excessive sexual behavior) among sexual offenders, the degree to which hypersexual behavior is linked to paraphilic and especially pedophilic interests in non-forensic populations has not been established. The purpose of the present study was to elucidate the associations between total sexual outlets (TSO) and other sex drive indicators, antisocial behavior, pedophilic interests, and sexual offending behavior in a large population-based community sample of males. The sample included 8,718 German men who participated in an online study. Hypersexual behavior as measured by self-reported TSO, self-reported sex drive, criminal history, and pedophilic interests were assessed. In moderated hierarchical logistic regression analyses self-reported contact sexual offending against children was linked to sexual fantasizing about children and antisociality. There was no association between aggregated sex drive, and sexual abusive behaviour in the multivariate analyses. In contrast, self-reported child pornography consumption was associated with sex drive, sexual fantasies involving children, and antisociality. Nevertheless, in convicted sexual offenders antisociality, sexual preoccupation (like hypersexuality), and pedophilic interest are important predictors of sexual reoffending against prepubescent children. Therefore, in clinical practice an assessment of criminal history and pedophilic interests in hypersexual individuals and vice versa hypersexuality in antisocial or pedophilic men should be considered.
